# Development and Characterization of Probiotic Beers with *Saccharomyces boulardii* as an Alternative to Conventional Brewer’s Yeast

**DOI:** 10.3390/foods12152912

**Published:** 2023-07-31

**Authors:** Ana Belén Díaz, Enrique Durán-Guerrero, Sergio Valiente, Remedios Castro, Cristina Lasanta

**Affiliations:** 1 Chemical Engineering and Food Technology Department, Faculty of Sciences-IVAGRO, University of Cadiz, Agrifood Campus of International Excellence (CeiA3), Polígono Río San Pedro, s/n, Puerto Real, 11510 Cadiz, Spain; anabelen.diaz@uca.es (A.B.D.); sergio.valientevelez@alum.uca.es (S.V.); cristina.lasanta@uca.es (C.L.); 2 Analytical Chemistry Department, Faculty of Sciences-IVAGRO, University of Cadiz, Agrifood Campus of International Excellence (CeiA3), Polígono Río San Pedro, s/n, Puerto Real, 11510 Cadiz, Spain; remedios.castro@uca.es

**Keywords:** probiotic, beer, *Saccharomyces boulardii*, volatile compounds, sensory analysis

## Abstract

The development of new non-dairy probiotic foods is interesting, given lactose intolerance, milk allergies, and the growing trend of vegetarianism. In this paper, beer has been used as a probiotic delivery matrix, using *Saccharomyces boulardii* as an alternative to conventional brewer’s yeast. The strain was able to grow in worts prepared with hops containing different alpha-acid concentrations, attaining in all cases a final cell concentration above 1·10^8^ cells mL^−1^. Some differences were found in the physicochemical parameters of beers brewed with *S. boulardii* compared to those brewed with a standard brewer’s yeast. Probiotic beers turned out to be less cloudy, which could help with a possible filtering step; less alcoholic in some cases; a healthier alternative; and with a slightly lower pH, interesting for the reduction of spoilage risk. Thirty volatile compounds were determined in the samples, and, in general, the beers brewed with the probiotic yeast presented significantly higher concentrations for the majority of the studied volatile compounds. In addition, multivariate statistical analysis was successfully performed to differentiate the beers obtained in terms of their volatile composition. Probiotic and standard beers were also subjected to sensory analysis, and they presented similar results in their overall impression.

## 1. Introduction

There is a growing demand by consumers for functional foods to help prevent chronic illnesses like cardiovascular disease, Alzheimer’s disease, and osteoporosis or to improve health and welfare [1]. This interest has driven the development and innovation of functional foods, making it necessary to search for new health-promoting bioactive compounds, including phytochemicals or herbs, natural antioxidants, probiotics, prebiotics, bioactive peptides, etc. [2]. Functional food is defined as food products with an added health benefit over and above their nutritional value [3]. Among them, the development of probiotic-based food is the fastest growing, representing 70% of the functional food market [4].

According to the Food and Agricultural Organization of the United Nations and the World Health Organization, reviewed by the International Association for Scientific Prebiotics and Probiotics (ISAPP), probiotics are “live microorganisms that, when administered in adequate amounts, confer a health benefit on the host” [5,6]. Probiotics release antibacterial substances or metabolites, such as lactic acid, preventing the growth of pathogens, activating the host immune response, and improving epithelial barrier function [7,8,9]. Lactic acid bacteria are the main probiotic group; Lactobacilli, like *Lactobacillus acidophilus* and *Lactobacillus rhamnosus*, and Bifidobacteria are the most commonly used [10,11]. Moreover, a few non-lactic microorganisms have been used, as in the case of the yeast *Saccharomyces cerevisiae* var. *boulardii,* which has been demonstrated to be an effective probiotic. This yeast has been shown to be a preventive and therapeutic agent for diarrhea and other gastrointestinal disorders produced as a consequence of antimicrobial agent administration [10]. Furthermore, other healthy properties, including antibacterial, antiviral, antioxidant, anticarcinogenic, anti-inflammatory, immunomodulatory, etc., have been clinically demonstrated [12].

Generally, dairy products have been used as carriers of probiotics, as they represent a favorable culture medium for their viability. However, given the high levels of lactose intolerance in the world population (≈65%), other illnesses associated with the consumption of these products (allergies against milk protein or to the presence of cholesterol), and modern lifestyles distant from animal food consumption, there is an increase in the use of nondairy foods for this purpose [13,14].

Beer takes the third position among the most popular drinks consumed in the world, after tea and coffee [15]. An increasing number of beer consumers eschew mass-produced lager brands and demand genuine, singular, and different high-quality products. For this reason, craft beer brewers are challenging lager brand producers, looking for product innovation (unique sensory features, original brewing styles, and selection of raw materials), sometimes outside of the beer industry for inspiration, which also provides them with higher economic benefits [16,17]. Given the rising consumer health awareness, there are several papers on functional beers, such as estrogenic, kefir, xanthohumol, and probiotic beers, among others [17,18,19]. Regarding this, beer represents a natural source of prebiotics, given the presence of high-molecular-weight pentosans and a mixture of high- and low-molecular-weight β-glucans, which can selectively stimulate the growth of probiotics resident in the gut [20]. For these reasons, craft beers could be an interesting carrier matrix for probiotics. However, maintaining the viability of the probiotic strain in the harsh conditions of beer is a major technological challenge. The microorganism used must show resistance to bitter iso-alpha acids, potent antimicrobial constituents that are extracted from hops during wort boiling. Probiotic lactic acid bacteria have been used in published papers for the development of this type of beer. However, they have been shown to be more susceptible to hop iso-alpha acids, in addition to producing sour beers. In this work, we have solved these problems by using a probiotic yeast strain that has shown some resistance to iso-alpha acids produced during wort boiling, which is interesting for the development of bitter probiotic beers.

Given that there are only a few studies on alcoholic beverages as a probiotic matrix, this study aimed to evaluate the potential use of craft beer as a carrier of the probiotic yeast *Saccharomyces boulardii*, evaluating its growth, viability, and stability during primary fermentation and at the end of secondary fermentation, using four hop varieties with different alpha-acid concentrations. Physico-chemical, aromatic, and sensory characterizations of elaborated beers were also performed.

## 2. Materials and Methods

Eight different craft beers were brewed, four of them with the probiotic yeast *Saccharomyces boulardii* and another four using a standard brewer’s yeast, *Saccharomyces cerevisiae,* using four different hop varieties for each one, two with a low content of alpha acids and two with a high content of these compounds.

### 2.1. Inoculum Preparation

Probiotic pure cultures of the probiotic yeast *Saccharomyces boulardii* (*Saccharomyces cerevisiae* Meyen ex E.C. Hansen 1883, CECT1474) were propagated in a medium containing 10 gL^−1^ glucose, 5 gL^−1^ mycopeptone, 3 gL^−1^ yeast extract, and 3 gL^−1^ malt extract at 26 °C for 72 h. The cultures, with viability above 90% and a cell count >10^8^ cells mL^−1^, were maintained as frozen stocks in 50% glycerol at −70 °C.

The freeze-dried non-probiotic yeast *Saccharomyces cerevisiae* (Lallemand Brewing) was used to be compared with the probiotic strain. For primary fermentation, 1.5 g of dry yeast was hydrated in 14 mL of tap water at 30 °C for 15 min.

### 2.2. Beer-Making Procedure

#### 2.2.1. Primary Fermentation

Commercial spray malt light extract (Muntons, Stowmarket, UK) was used for sweet wort preparation, which was composed of 100% barley malt with a protein content of 7.5, a pH of 5–6, and an EBC of 7–12. For this purpose, the extract was reconstituted with 8 L of pure drinking water per kg of solids. Four different hops from Laguilhoat (Fuenlabrada, Spain) were used separately in the boiling stage: two with low content in alpha acids, Crystal (4.9%) and Bobek (3.5%), and two with a high content of these compounds, Polaris (20.1%) and Columbus (16.2%). The boiling was carried out in a BrauEule II (Brumas, Bayrischzell, Germany) brewing equipment at 100 °C for 90 min by using 1.5 gL^−1^ of pellet hops. It was a 34-L stainless steel equipment (a closed boiler that works by generating steam) with programmable temperature control, equipped with a whirlpool system, and automatic cleaning. Dimensions (W, T, D): 60 cm, 57 cm, 50 cm. After the boiling stage, the trub was removed by a whirlpool, and the wort obtained was tempered at 15 °C and adjusted to an original gravity of 11 Plato degrees (°P) before fermentation with pure drinking water.

For primary fermentation, 1.8 L of hopped wort was inoculated with 10^6^ cells mL^−1^ of yeast, probiotic or non-probiotic, which was incubated statically in a 2 L glass flask in a thermostat cabinet at 20 °C for 9 days. Each experiment was carried out in duplicate.

During fermentation, samples were taken periodically for the analysis of yeast cell counting and viability.

#### 2.2.2. Secondary Fermentation and Maturation

Beer was carbonated naturally through secondary fermentation by adding sucrose to allow the remaining yeast to produce additional CO_2_. For this purpose, the broth after primary fermentation was divided into two 750 mL fractions and introduced into a 1 L brown beer bottle with a swing stopper. It was calculated that the amount of sucrose needed to produce 2.6 gL^−1^ CO_2_ at 20 °C was added to each bottle. Bottle fermentation and maturation were carried out for 30 days, at which point the beers were transparent and most of the particles had settled out.

### 2.3. Yeast Cell Counting and Viability

Yeast cell counting during primary fermentation and at the end of secondary fermentation was performed in a Neubauer chamber. For cell viability, the cell counting was performed after mixing the samples with an equal volume of 0.01% *w*/*v* methylene blue solution, which selectively stains dead cells.

### 2.4. Physical-Chemical Analysis

Alcohol content and color (°EBC) were analyzed following the official analytical method of the Analytical Division of the European Brewery Convention [21,22], using an Anton Paar densimeter model DMA 500 and a Genesys 10 UV spectrophotometer, respectively.

The gravity level in Plato degrees (°P) was determined in a 0−10 scale densimeter calibrated at 20 °C (Alla France, Chemillé en Anjou, France). Regarding pH, it was measured using a pH meter (Hach, Ames, IA, USA).

The turbidity of beers was measured in a turbidimeter (Hach), which gives the results in nephelometric turbidity units (NTU).

For bitterness determination, it was followed by the ASBC method Beer-23A.

Regarding the total phenolic index (TPI) of beers, samples were measured at 280 nm in a spectrophotometer after 1:40 *v*/*v* dilution.

For color, TPI, and bitterness determination, samples were previously filtered using a nylon filter (0.45 µm, RephiQuik, Madrid, Spain).

Each analysis was carried out in duplicate.

### 2.5. Analysis of Volatile Compounds

Volatile compounds in samples were analyzed through gas chromatography with mass spectrometry detection and previous stir bar sorptive extraction (SBSE) [23]. Briefly, 50 mL of beer, with 25% NaCl (*w*/*v*) added, were extracted for 180 min at 1000 rpm employing PDMS stir bars (Gerstel, Mülheim an der Ruhr, Germany). After the extraction procedure, the stir bars were thermally desorbed in a thermal desorption unit (TDS-2, Gerstel) for their later chromatographic analysis. An Agilent 6890 GC (Agilent Technologies, Palo Alto, CA, USA) was employed, equipped with a DB-Wax (J&W Scientific, Folsom, CA, USA) capillary column (60 m × 0.25 mm I.D., 0.25 μm coating) coupled to an Agilent 5973 MS detector (Agilent Technologies). Each analysis was performed in duplicate.

### 2.6. Sensory Analysis

The sensory evaluation of beers was carried out in a tasting room according to ISO 8589. For this purpose, it was selected a sensory panel of 10 experts (6 women and 4 men), with a range age of 30–62 years and extensive experience in food sensory evaluation, four of whom were members of official tasting panels (P.D.O. Sherry Wine and P.D.O. Sherry Vinegar). Moreover, the panelists were trained to evaluate the appearance and aroma profiles of beers.

Sensory analysis of the beers was conducted in two sessions, evaluating four products in each session and in duplicate form, which were identified by a three-digit code. Samples were served at 10 °C in odorless and colorless 100 mL glass cups, which were covered with a watch glass to avoid evaporation of volatile compounds.

In the descriptive sensory analysis, it was analyzed specifically for aroma, evaluating the descriptors cereal, green fruit, tropical fruit, citrus fruit, stone fruit, floral, herbaceous, vegetables, caramel, chocolate, and toast using a numerical scale from 1 (low intensity) to 5 (high intensity), with 0 being the absence of that aroma descriptor. Finally, an overall rating of the quality of beers was provided by the panelists using a five-point scale based on their appearance and aroma.

### 2.7. Statistical Analysis

Data were analyzed using Statistica V12.5 software (StatSoft GmbH, Hamburg, Germany) and Statgraphics Centurion, Version 15.0 (Statpoint Inc., Warrenton, VA, USA). Specifically, analysis of variance (ANOVA), Tukey test, cluster analysis (CA), and principal component analysis (PCA) were performed.

## 3. Results and Discussion

### 3.1. Yeast Cell Counting and Viability

**Figure 1:**
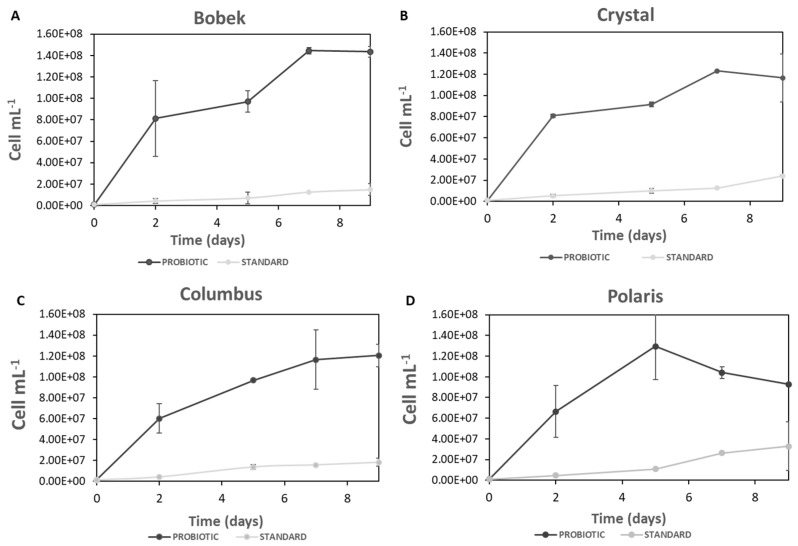
Yeast cell counting during fermentation with standard and probiotic yeasts with the four hops tested ((**A**): Bobek, (**B**): Crystal, (**C**): Columbus, and (**D**): Polaris).

**Figure 2:**
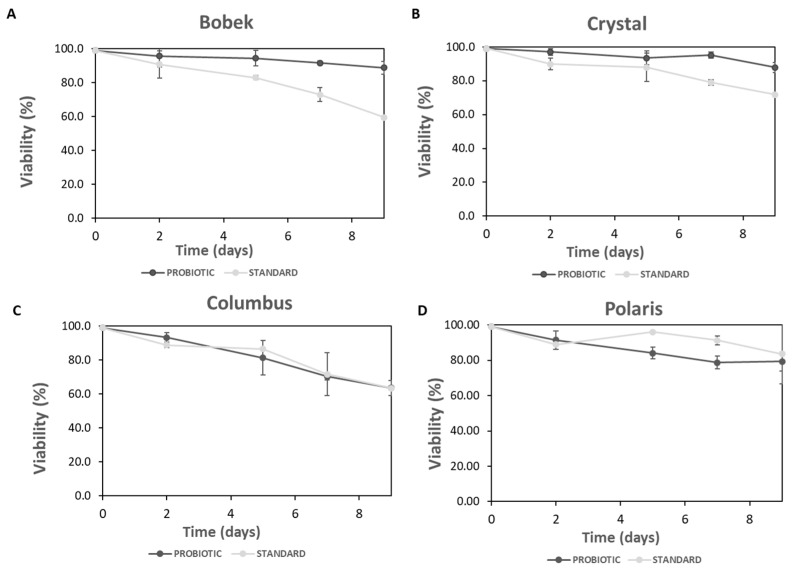
Yeast cell viability during fermentation with standard and probiotic yeasts with the four hops tested ((**A**): Bobek, (**B**): Crystal, (**C**): Columbus, and (**D**): Polaris).

Yeast cell counting of standard and probiotic yeasts during primary fermentation with the four different hops (Bobek, Crystal, Columbus, and Polaris) was determined. As shown in Figure 1, in all cases, the cell concentration of standard yeast increased with the fermentation time. It seems that the content of alpha acids in hops did not influence cell concentration, as 1.5·10^7^ and 2.4·10^7^ cells mL^−1^ were counted at the end of fermentation with Bobek and Crystal, respectively (with low alpha acid content), and 1.8·10^7^ and 3.2·10^7^ cells mL^−1^, with Columbus and Polaris, respectively (with high alpha acid content). Regarding the viability of the standard yeast (Figure 2), it decreased during fermentation with all the studied hops, with this decrease being more significant for Bobek, Crystal, and Columbus, with final values of 60, 70, and 60%, respectively. A slight decrease was observed with Polaris, keeping viability above 80% on the ninth day of fermentation.

In fermentation with Bobek, the concentration of probiotic cells increased sharply the first 7 days of fermentation and kept constant till the ninth day, with a final cell concentration of 1.5·10^8^ cells mL^−1^. In the case of Crystal, also with low alpha-acid content, there was a marked increase in cell concentration in the first 2 days, and then it continued to grow during the whole fermentation, achieving a final concentration of 1.2·10^8^ cells mL^−1^. By using these hops with a low content of alpha acids, the viability of the cells remained above 95% at the end of fermentation.

Similar behavior was detected with Columbus, with a high alpha acid concentration (16.2%), increasing cell counting till the end of the process with a final value of 1.2·10^8^ cells mL^−1^. However, a fall in cell viability was also observed during the fermentation, reaching a value of 60% on the 9th day of fermentation. As regards Polaris hops, cell counting increased until day 5, after which the decline of cells was observed. At the end of fermentation, still, 1.0·10^8^ cells mL^−1^ were counted, with cell viability above 80%.

Similar final concentrations of the probiotic cells were achieved regardless of alpha acid concentration in hops, with values varying from 1.0–1.5·10^8^ cells mL^−1^, and viabilities above 80% in all cases, with the exception of Columbus with 60% viability. These values were much higher than those achieved with the standard yeast with the four hops tested, with final cell concentrations in the range of 1.5–3.2·10^7^ cells mL^−1^ and viabilities below 80% for Bobek, Crystal, and Columbus. The concentration of probiotic cells at the end of fermentation in this work was higher than that achieved by Capece et al. [24] (8.0·10^6^−7·10^7^ viable cells mL^−1^) in a mixed fermentation with an *S. cerevisiae* strain.

It can be concluded that the probiotic yeast is grown in worts prepared with the four hops tested, containing different alpha acid concentrations, with an average cell count at the end of fermentation above 1·10^8^ cells per mL^−1^. For this strain, lower cell viability was attained with hops containing high alpha acids; however, in all cases, a value above 60% at the end of fermentation. Moreover, higher initial growth rates were achieved with hops containing low alpha acids, specifically 2.07·10^6^ and 1.67·10^6^ cells h^−1^ with Bobek and Crystal, respectively, compared to 1.40·10^6^ and 1.12·10^6^ cells h^−1^, calculated for Columbus and Polaris, respectively. A decrease in the specific growth rate of *S. cerevisiae* var. *boulardii* was observed by Senkarcinova et al. [17] at 50 IBU, compared to 15 and 30 IBU, in probiotic alcohol-free beers. As can be seen in our study, probiotic yeast was more adapted to the wort compared to the standard *S. cerevisiae* yeast, which is an additional advantage of this alternative strain for the production of beer.

The high resistance of this yeast species to iso-alpha acids produced during wort boiling with hops compared to other probiotic microorganisms is promising. It indicates that it might be possible to obtain probiotic beers with different bitterness levels by using hops with different alpha acid concentrations. These compounds have been shown to be potent antimicrobial compounds that inhibit probiotic lactic acid bacteria. In fact, these bacteria can be affected by harsh conditions in beer, including ethanol (normally 3–5%), acidity (pH 3.9–4.4), elevated carbon dioxide, a lack of nutrients, and hop iso-alpha acids (17–55 ppm) [25].

Several lactic acid bacteria have been used to produce probiotic beer, like *Lactobacillus paracasei*, *L. acidophilus*, *L. rhamnosus*, *L. bulgaricus*, etc. [17,26]. In some of these works [26], no viable cells of these bacteria were detected after 7 days of co-fermentation with *S. cerevisiae*.

Lactic acid bacteria are also used to produce sour beer (beer with an intentionally sour taste). Given their growth inhibition with iso-alpha acids, they are limited to 25 IBUs as a maximum bitterness level [27]. However, to produce other probiotic beer styles with higher concentrations of alpha acids (including lagers, stouts, IPAs, etc.), more tolerant microorganisms are needed. It has been reported that yeasts show little or no inhibition toward them [28]. In this regard, the probiotic yeast *Saccharomyces boulardii* has been used to develop probiotic wheat beer, demonstrating survival after in vitro gastrointestinal transit [29].

### 3.2. Beer Characterization after Secondary Fermentation and Maturation

#### 3.2.1. Yeast Cell Counting and Viability

**Figure 3:**
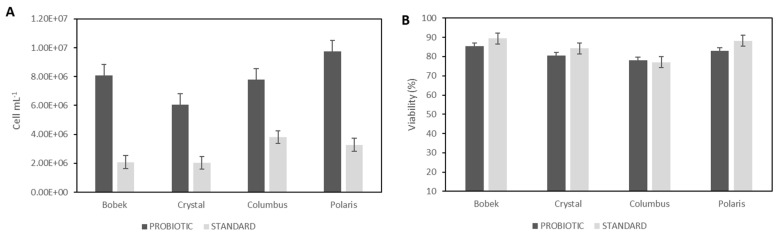
Cell counting (**A**) and viability (**B**) of probiotic and standard yeasts after 30 days of secondary fermentation and maturation using the four hop varieties (Bobek, Crystal, Columbus, and Polaris).

The cell count of probiotic and standard yeasts was evaluated after secondary fermentation and maturation in bottles. As can be seen in Figure 3A, there was not a negative correlation between the content of alpha acid in hops and the number of standard and probiotic yeast cells. In fact, the highest cell concentrations were 3.8·10^6^ and 9.8·10^6^ cells mL^−1^ for the standard and probiotic yeast were achieved with Columbus and Polaris, respectively, which contain a high concentration of alpha acids.

As can be seen in Figure 3A, the concentration of cells was significantly higher in beers with probiotic yeast. Cell concentration varied from 2.0·10^6^ to 3.8·10^6^ cells mL^−1^ using standard yeast and from 6.1·10^6^ to 9.8·10^6^ cells mL^−1^ with *Saccharomyces boulardii*, with cell viability above 77% in all cases. The alcohol content of probiotic beers was slightly lower in some cases compared to beers obtained with standard yeast. Perhaps this lower conversion of alcohol influenced a higher growth of the probiotic yeast in the initial stages of beer production, and therefore, a higher content was found in the final beer. In other studies, *S. boulardii* has demonstrated higher viable cells than *S. cerevisae* after 36 days of beer refrigerated storage [30]. Moreover, a concentration of viable cells above 1·10^7^ cfu mL^−1^ was detected after in vitro gastric treatment of wheat beer with this yeast after a storage period of 30 days at 0 °C and an in vitro gastric treatment.

It is very important to maintain the viable count of the probiotic microorganisms in the final product until it is consumed [31]. In general, it has been accepted that a minimum level of 10^6^ cells mL^−1^ and it is recommended that a daily intake of probiotic products contain approximately 10^9^ viable cells [32]. Thus, all the beers produced with *S. boulardii* in this work, using four hops with different alpha acid concentrations, can be considered probiotics. Considering the viable cells at the time of bottle opening and the recommended intake of probiotics, a daily intake of 145, 205, 165, or 123 mL of beers with Bobek, Crystal, Columbus, and Polaris seems appropriate.

#### 3.2.2. Physicochemical Parameters

The average values of the physicochemical parameters measured after the second fermentation in the different final beers are presented in Table 1. In general, it can be seen that beers produced with the probiotic strain showed similar physicochemical characteristics to those brewed with the standard one.

Plato’s degree was measured after the second fermentation. A lower dry extract content in the standard yeast was observed, with the exception of Columbus hops. These results indicate a higher consumption of sugars in the standard strain compared with the probiotic one. However, significant differences were only observed between both strains when using Crystal hops.

As can be seen, the yeast strain has a certain influence on the turbidity, showing higher values for the standard yeast, being above 30 NTU in all cases, and attaining the maximum value of 63.40 NTU in the beer brewed with hops of the variety Columbus. On the contrary, beers brewed with the probiotic strain showed lower turbidities, within a range of 13.50 and 28.00 NTU, which can be a technological advantage in order to facilitate the subsequent filtering. Turbidity in the final beers is affected by the growth of yeast and cell sedimentation speed. Furthermore, yeast metabolism during fermentation can affect haze formation [33].

The alcohol content of beers ranges from 3 to 14% *v*/*v* by normal fermentation, although the common styles do not exceed 6% [34]. Moreover, normally, craft beers tend to have a higher alcohol content than industrial ones [35]. In this work, beers obtained with standard yeast showed an alcohol content within the range of 4.90 and 3.98% *v*/*v*, being slightly lower with the probiotic beers for all varieties of hops used, specifically between 4.67 and 3.26% *v*/*v*. However, only significant differences were observed for Crystal hops. These results agree with others obtained in previous studies [30]. This fact might be interesting in order to produce healthier beers with a lower alcohol degree and probiotics, given that the demand for beers with these characteristics represents a fast-growing segment in the global beer market. For this purpose, the use of *Saccharomyces boulardii* is interesting, as it acts as a probiotic yeast that also tends to produce beers with less alcohol [36]. Similar alcohol content was assayed in a wheat beer brewed with the same probiotic yeast, specifically showing a value of 4.01% *v*/*v* [30]. However, a higher value (6.06% *v*/*v*) showed the probiotic beer was also brewed with *S. boulardii* in a Pilsen-type wort [37].

Regarding pH, its value in beers usually ranges from 3.9 to 4.5, affecting their stability against microbial spoilage, colloidal, foam, and flavor stability, drinkability, and palate smoothness [38]. In the present work, the pH value was within this range in most cases, being slightly lower with the probiotic strain compared with the standard one (between 4.29–4.42 versus 4.39–4.81), with no significant differences for Bobek hops. This can be related to the greater viability and growth of the probiotic strain, and it could be considered an advantage in order to prevent spoilage risk [17]. Moreover, this advantage is useful for the production of craft beers on a large scale. These results agree with those of Mulero-Cerezo, who obtained higher acidification with the probiotic yeast, reducing the contamination risk of produced beers [30].

Regarding bitterness, this parameter is correlated with the variety of hops used in the brewing process, which have a low or high content of alpha acids. The bitterness of beers is frequently measured in IBUs, which typically range between 5 and 120, and are considered high bitterness with values over 25 [39,40]. Columbus hops are generally used as bittering hops [41]. In this work, beers brewed with Columbus and Polaris hops, with a high content of alpha acids, showed higher International Bitterness Units (IBU), rating between 37.28 and 40.73, unlike those brewed with Crystal and Bobek, with values within the range of 13.15 and 17.95. Comparing bitterness values in beers produced with standard and probiotic beers, they were higher in the former when using Columbus and Polaris; however, no significant differences were observed for Crystal and Bobek.

As for color, all of them are referred to as “pale beers,” with EBC values below 18. In this work, probiotic beers showed slightly lower EBC values, ranging between 12.45 and 17.19, than standard ones, with values within the range of 14.14 and 17.81. However, no significant differences were observed comparing both strains, with the exception of Crystal hops. Beers brewed with hops of the variety Bobek showed the highest EBC values for both strains.

Finally, polyphenolic content was assayed in the brewed beers. Polyphenols generally come from malt and hops, with the latter responsible for around 30% of TPI in beers [42]. In this work, the highest TPI value of 29.72 was measured in the beer brewed with Columbus hops using the probiotic strain. In general, the TPI of probiotic beers was higher than that of standard ones. However, only significant differences were observed with Columbus hops. This fact is interesting considering that polyphenol content is associated with a greater antioxidant capacity. Polyphenols already identified in beers have turned out to have antioxidant, anti-inflammatory, estrogenic, and anti-carcinogenic activities [43,44,45]. Normally, craft beers show higher antioxidant activity compared with commercial ones. In general, filtration seems to be one of the main stages responsible for the drastic reduction of polyphenols during the brewing of industrial beers.

#### 3.2.3. Volatile Profile

Beer is a complex mixture containing numerous volatile compounds, which are responsible for beer flavor and can be derived from ingredients like hops and barley, roasting of malt, wort boiling, yeast or contaminants’ metabolism, process conditions, etc. [46]. These compounds include higher alcohols, esters, carbonyl compounds, fatty acids, furanic compounds, monoterpenols, C_13_-norisoprenoids, and volatile phenols, among others [47].

A total of 30 volatile compounds were quantified in the beers developed in the present work, mainly esters, alcohols, and acids. The concentration of these volatile compounds was subjected to a statistical analysis of variance in order to study the influence of the type of yeast (standard or probiotic) and the variety of hops (Table 2). Values marked with an asterisk indicate the presence of significant differences (*p* < 0.05). In general, it can be seen that the volatile compounds are influenced by the variables. However, it should be highlighted that the variable with the most significant influence on a large number of volatile compounds is the type of yeast used. Thus, quite significant differences in the volatile profile were observed by using standard or probiotic yeast; however, the hop variety affected only a few volatile compounds in a significant way.

Due to the higher influence of the type of yeast employed, in Table 3, mean values of the concentrations (μgL^−1^) of the volatile compounds quantified in beers produced with probiotic and standard yeasts are shown. The volatile compounds that presented higher concentrations in the samples were ethyl acetate, 3-methyl-1-butanol, furfural, isovaleric acid, hexanoic acid, phenethyl alcohol, nerolidol octanoic acid, and decanoic acid, which were found in ranges of mg L^−1^. It can be seen that the probiotic yeast produced a higher concentration of volatile compounds for the majority of compounds compared with the standard one. This is the case with some acids, such as hexanoic acid, octanoic acid, nonanoic acid, and decanoic acid. Many studies have reported the production of short-chain fatty acids by the action of probiotic microorganisms [48]. Some esters, characterized by fruity and floral aromas, such as ethyl heptanoate, ethyl octanoate, ethyl decanoate, or ethyl dodecanoate, were also found in higher concentrations in probiotic beers. These compounds are important aroma-active esters in beer, and are also present in beer with standard yeast [49,50]. Most of the esters have also been found in other beers made with *S. boulardii*. This is the case of the probiotic alcohol-free beer made by Senkarcinova et al. [17] by using this strain.

However, other important odorant compounds like ethyl acetate, 3-methyl-1-butanol, octanol, and isobutyric acid are present in higher concentrations in beers produced with standard yeasts. The result agrees with those obtained by Capece et al. [24], who produced a higher concentration of these volatile compounds by using *S. cerevisiae* than with *S. cerevisiae* var. *boulardii*. Other esters with fruity and floral aromas, like ethyl butyrate, ethyl isovalerate, isopentyl acetate, and phenethyl acetate, among others, also presented significantly higher concentrations in beers produced with standard yeasts (Table 3).

Canonico et al. [51] showed significant differences in the aroma profile of probiotic beers compared with traditional ones, using *L. thermotolerans* and *K. unispora*. However, other authors found similar concentrations of aroma compounds in beers with and without probiotic yeasts, employing *S. boulardii* [37].

In order to further study the volatile profile from a multivariate point of view, a Principal Component Analysis (PCA) was performed on the dataset, obtaining five groups with an eigenvalue greater than 1. Five components explained 88.892% of the variability of the samples; however, two principal components were sufficient to explain more than half of the variability of the samples (60.719%).

The compounds that contributed in a higher way to PC1 were 1-decanol, beta-damascenone, nerolidol, octanoic acid, decanoic acid, or ethyl decanoate, among others. PC2 presented a higher contribution of furfural, 2-propanone-1-hydroxy, ethyl acetate, ethyl isovalerate, isopentyl acetate, ethyl butyrate, or acetic acid, among others.

**Figure 4:**
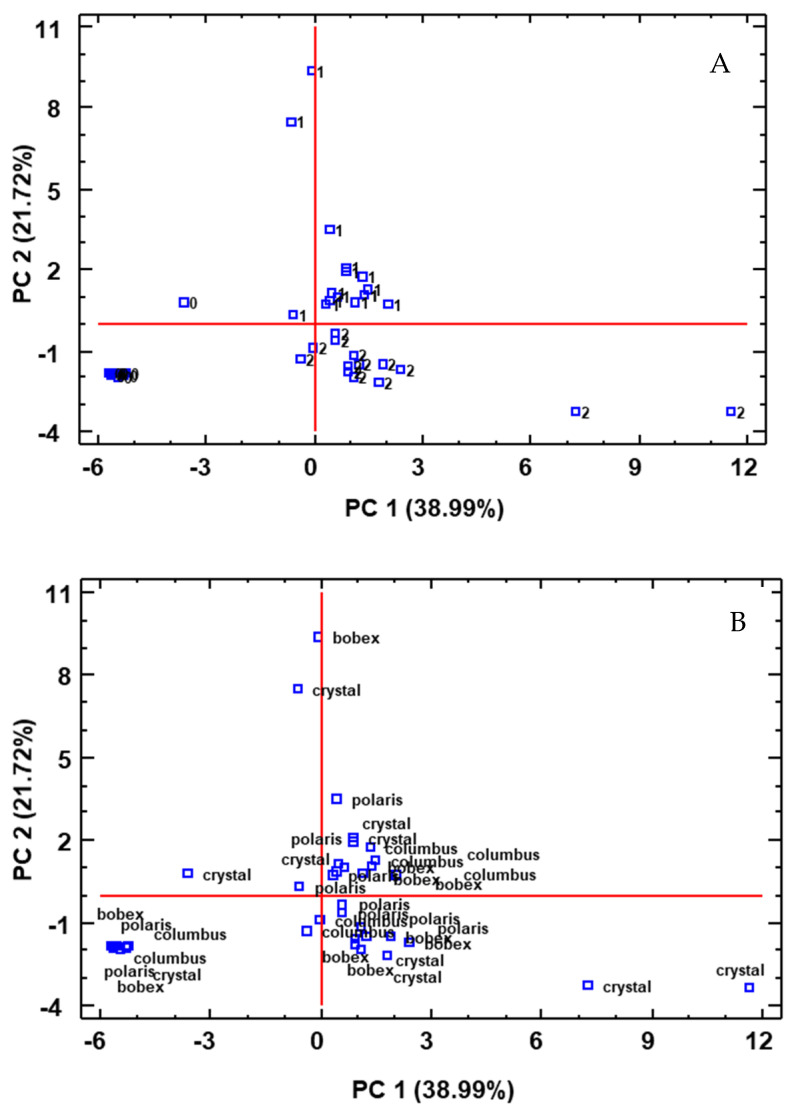
Distribution of all samples on the plane defined by the PCs as a function of the yeast used (**A**) and the variety of hop (**B**). 0: initial samples before inoculation; 1: beers produced with standard yeast; 2: beers produced with probiotic yeast.

Figure 4 shows the distribution of all the samples on the plane defined by PC1 and PC2, as a function of the yeast used and the variety of hops. In Figure 4A, the groups associated with the type of yeast can be seen. The negative values of both component 1 and 2 represented the initial wort before inoculation. Beers produced with standard yeast (labeled by 1) were situated in the first quadrant of the graph (positive values of PC1 and PC2), whereas probiotic beers were placed in quadrant 2 (negative values of PC2). Thus, the type of microorganism used in the fermentation was an important factor that affected the volatile profile of beers, as was previously shown with the analysis of variance. However, the distribution of all samples as a function of the variety of hops used (Figure 4B) showed that there was no correlation, so it seems that the type of hops was not an influential factor in the formation of volatile compounds.

**Figure 5:**
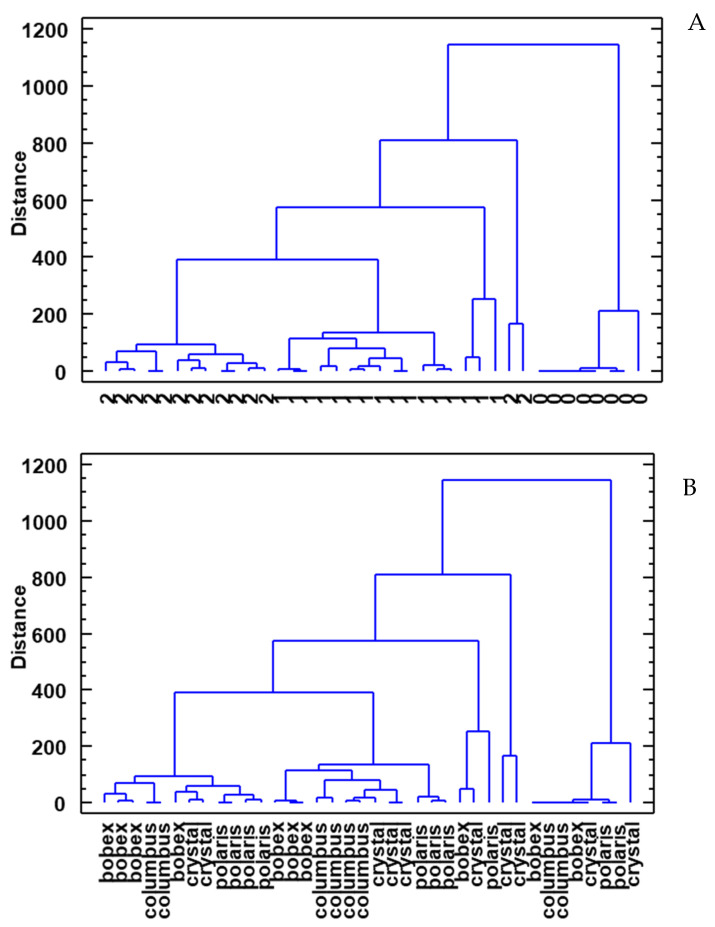
Cluster analysis as a function of the type of yeast (**A**) and the type of hops (**B**). 0: initial samples before inoculation; 1: beers produced with standard yeast; 2: beers produced with probiotic yeast.

Finally, a cluster analysis of the data obtained from the samples was carried out to graphically demonstrate the differences and similarities with respect to the volatile profiles of the beers (Figure 5). Three clusters can be differentiated in (A): beers made by standard yeast, probiotic beers, and initial samples before inoculation. These results again reflect the influence of the type of yeast on the volatile profile of beers. On the contrary, clear groups were not observed as regards the type of hops used (B).

#### 3.2.4. Sensory Analysis

It is difficult to translate the volatile composition of a beverage into an aroma. The influence of the matrix and the presence of other components play an important role in the perceived aroma [52]. On the other hand, several authors have noted the importance of certain parameters in the sensory characteristics of beers, with the hop variety and the yeast strain employed among the most important ones [53,54,55]. Although several studies have been carried out about the use of *S. Boulardii* for brewing, no sensory results have been found except that it does not negatively affect beer aroma [24] and that it would be well accepted by consumers in terms of preference [37].

**Figure 6:**
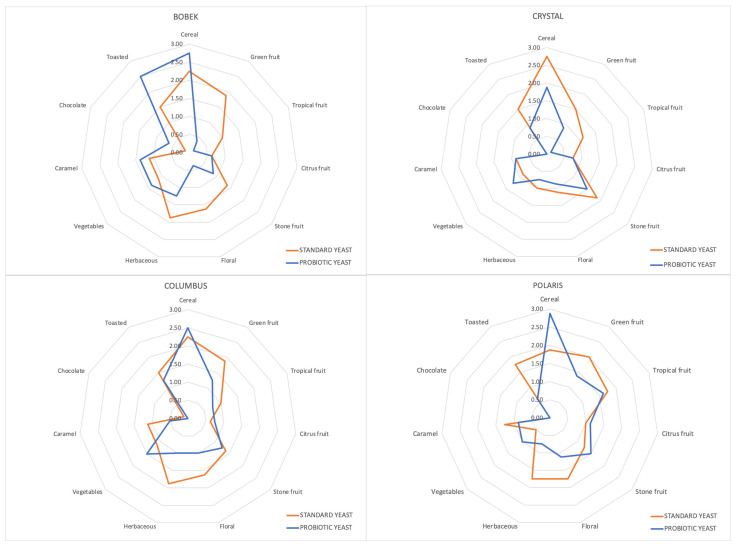
Descriptive analysis of beers produced with standard and probiotic beers using the four hop varieties.

In this study, eleven descriptors were evaluated for beers brewed with control and probiotic yeast for all hop varieties in order to describe their aroma. In Figure 6, the punctuation of all of them in the descriptive tasting is represented when comparing both types of beer with each hop variety. As expected, with the same fermentation conditions and the same raw materials except for the hop variety, the aromatic profile changed. Furthermore, as can be seen, the yeast employed in the fermentation stage modified the organoleptic profiles of the different beers. This is in consonance with the differences observed in the volatile composition of the different strains. These modifications were different according to the employed hop variety. In general, more fruity and floral characters were described in the beers elaborated with standard yeast. They also obtained better punctuation in the herbaceous character. On the other side, higher punctuation was obtained in the cereal descriptor for the beers elaborated with the probiotic strain in three of the four hop varieties.

**Figure 7:**
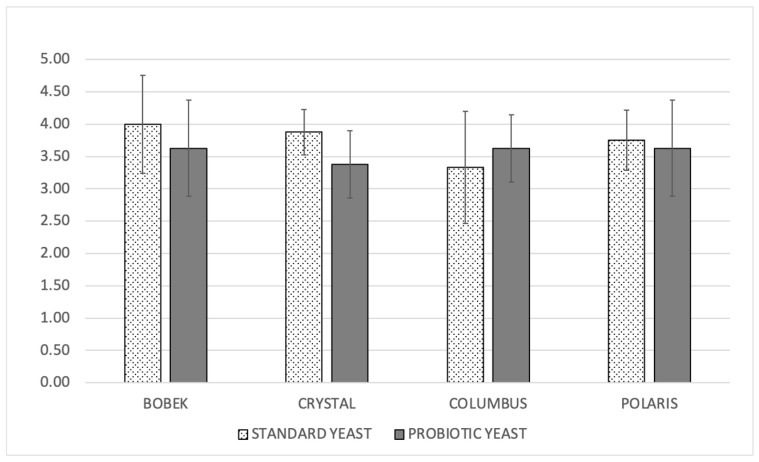
Overall quality of produced beers on a 1–5 scale.

To evaluate the overall impression, a discrete scale of five points was used. As can be seen in Figure 7, all the beers were scored between 3 and 4 points, and the beers elaborated with the control yeast were in general better evaluated than the probiotic ones, although with small (and not significant) differences. These first results are positive because, although the use of *S. boulardii* changed the aromatic profile of the elaborated beers, no off-flavors were detected, and the overall impression was similar to that of beer elaborated with standard yeast strains.

## 4. Conclusions

*Saccharomyces boulardii* represents a promising alternative to conventional brewer’s yeast for the production of probiotic beers. Cell concentration after 30 days of second fermentation and maturation was above 6.1·10^6^ cells mL^−1^ with this strain, with cell viability above 77% in all cases when using four hops with different alpha acid concentrations. The physicochemical parameters of probiotic and standard beers were similar. However, some benefits can be associated with probiotic beers. They showed lower turbidities, which could facilitate the subsequent filtering, and a lower pH, preventing the spoilage risk. Moreover, the probiotic strain produced beers with a slightly lower alcohol content. This fact, together with the presence of probiotics, is interesting for the development of healthier beers. In general, a higher concentration of volatile compounds was found in beers brewed with *S. boulardii,* demonstrating that the type of microorganism was the most influential factor that affected the volatile profile rather than the type of hops. The sensory analysis of the brewed beers showed a different aromatic profile depending on the yeast strain, with a more intense cereal aroma in probiotic beers and fruitier and more floral aromas in standard ones. No off-flavors were detected in any of the elaborate beers.

## Figures and Tables

**Table 1:** Physicochemical parameters of probiotic and standard yeasts using the four hop varieties (Bobek, Crystal, Columbus, and Polaris).

	Bobek		Crystal		Columbus		Polaris	
	Standard	Probiotic	Standard	Probiotic	Standard	Probiotic	Standard	Probiotic
°P	3.50 ± 0.71	4.75 ± 0.35	3.00 ± 0.00 *	5.50 ± 0.71 *	4.75 ± 0.35	4.15 ± 0.21	2.75 ± 0.35	4.25 ± 0.35
Alcohol **(% *v*/*v*)**	4.19 ± 0.58	3.72 ± 0.49	3.98 ± 0.05 *	3.26 ± 0.15 *	4.90 ± 0.32	4.67 ± 0.57	4.63 ± 0.16	3.75 ± 0.47
NTU	52.05 ± 1.34 *	15.50 ± 4.24 *	40.95 ± 3.04	28.00 ± 3.54	63.40 ± 9.33 *	16.90 ± 1.27 *	33.10 ± 1.56 *	13.50 ± 1.41 *
pH	4.39 ± 0.03	4.33 ± 0.01	4.81 ± 0.06 *	4.42 ± 0.04 *	4.74 ± 0.01 *	4.33 ± 0.09 *	4.39 ± 0.01 *	4.29 ± 0.01 *
Biterness (°IBU)	17.95 ± 0.57	17.63 ± 0.18	14.63 ± 2.09	13.15 ± 0.64	40.73 ± 0.88 *	46.03 ± 0.46 *	38.23 ± 0.04 *	37.28 ± 0.11 *
Color (EBC)	17.81 ± 0.02	17.19 ± 0.55	14.14 ± 0.12 *	12.45 ± 0.35 *	16.24 ± 0.34	15.90 ± 0.25	15.51 ± 0.30	14.94 ± 0.30
TPI	23.08 ± 0.74	28.46 ± 1.67	21.56 ± 0.57	21.62 ± 0.48	22.02 ± 0.08 *	29.72 ± 0.11 *	23.50 ± 0.42	22.64 ± 0.57

* These values show statistically significant differences between standard and probiotic yeast (ANOVA, *p* < 0.05).

**Table 2:** ANOVA of volatile compounds identified for the variables type of yeast and hop variety.

Compounds	Type of Yeast		Hop Variety	
	F-Ratio	*p*-Value	F-Ratio	*p*-Value
Ethyl acetate	5.6636	0.0244 *	0.4811	0.6983
Ethyl butyrate	10.4308	0.0032 *	0.3434	0.7942
Ethyl isovalerate	7.1681	0.0123 *	0.5626	0.6445
Isopentyl acetate	7.6321	0.0100 *	0.3396	0.7968
3-methyl-1-butanol	6.2940	0.0182 *	0.1527	0.9270
Ethyl hexanoate	0.7702	0.3876	1.0119	0.4033
2-propanone-1-hydroxy	0.9707	0.3329	0.5147	0.6757
Ethyl heptanoate	10.4711	0.0031 *	6.0744	0.0028 *
Acetic acid	0.0297	0.8644	1.8376	0.1651
Ethyl octanoate	6.4916	0.0166 *	0.5152	0.6755
Heptanol	18.5759	0.0002 *	0.7303	0.5433
Furfural	0.4046	0.5299	1.8836	0.1571
Benzaldehyde	1.9839	0.1700	2.1051	0.1240
Linalool	3.4916	0.0722	9.3875	0.0002 *
Octanol	5.7865	0.0230 *	3.3051	0.0358 *
Isobutyric acid	20.7668	0.0001 *	0.0818	0.9693
Ethyl decanoate	10.1045	0.0036 *	2.0291	0.1344
Isovaleric acid	0.4728	0.4974	1.4605	0.2483
Hexanoic acid	12.1402	0.0016 *	1.4144	0.2610
1-decanol	1.1710	0.2884	6.4809	0.0020 *
Phenethyl acetate	30.3246	0.0000 *	0.1266	0.9435
Beta-damascenone	-	-	-	-
Ethyl dodecanoate	11.8227	0.0018 *	2.4504	0.0860
Benzenepropanoic acid ethyl ester	8.5046	0.0069 *	1.9490	0.1465
Phenethyl alcohol	3.3308	0.0787	0.1240	0.9451
Nerolidol	19.2300	0.0001 *	1.7629	0.1790
Octanoic acid	7.6275	0.0100 *	2.1012	0.1245
Nonanoic acid	19.4279	0.0001 *	1.3467	0.2809
2-methoxy-4-vinylphenol	1.2870	0.2662	4.0242	0.0178 *
Decanoic acid	32.8046	0.0000 *	1.6548	0.2012

* Significant differences (*p* < 0.05).

**Table 3:** Mean concentrations of volatile compounds in beers (μg L^−1^) for the four hops used in probiotic and standard beers.

Compounds	Probiotic		Standard	
	Mean	SD	Mean	SD
Ethyl acetate *	13.929	11.740	68.276	84.553
Ethyl butyrate	191.878	50.994	433.596	275.350
Ethyl isovalerate	45.737	27.852	206.499	222.667
Isopentyl acetate	360.246	113.297	1684.134	1790.154
3-methyl-1-butanol *	45.341	13.006	76.379	44.571
Ethyl hexanoate	304.747	177.626	261.192	83.581
2-propanone-1-hydroxy	429.688	544.125	762.039	1011.024
Ethyl heptanoate	11.346	3.533	8.036	1.942
Acetic acid	148.546	345.913	132.969	218.214
Ethyl octanoate	613.146	437.235	318.279	144.892
Heptanol	93.060	44.933	35.879	26.469
Furfural *	1.812	2.699	1.318	1.437
Benzaldehyde	70.237	69.558	41.813	38.508
Linalool	16.995	4.439	13.934	4.509
Octanol	10.830	2.468	18.730	12.044
Isobutyric acid	122.773	132.467	518.174	299.536
Ethyl decanoate	130.420	118.812	34.908	18.690
Isovaleric acid *	6.813	9.461	4.990	4.513
Hexanoic acid *	3.130	1.609	1.683	0.399
1-decanol	5.788	2.762	6.913	2.908
Phenethyl acetate	92.186	42.099	274.397	117.149
Beta-damascenone	<LOQ	-	<LOQ	-
Ethyl dodecanoate	96.002	64.856	36.407	23.270
Benzenepropanoic acid ethyl ester	<LOQ	-	0.266	1.076
Phenethyl alcohol *	22.654	11.198	29.188	8.363
Nerolidol *	1.455	0.305	1.056	0.185
Octanoic acid *	5.702	3.784	3.035	0.761
Nonanoic acid	79.388	16.247	61.015	3.664
2-methoxy-4-vinylphenol *	13.149	7.888	9.903	7.757
Decanoic acid	380.558	114.268	183.172	72.370

* mg L^−1^; SD: standard deviation, <LOQ: below limit of quantification.

## Data Availability

Data is unavailable due to privacy or ethical restrictions.
